# Creating a centralized social media recruitment service for research teams at the University of Michigan

**DOI:** 10.1017/cts.2020.540

**Published:** 2020-09-11

**Authors:** Aalap Doshi, Lisa Connally, Anita Johnson, Abbey Skrzypek

**Affiliations:** Michigan Institute for Clinical and Health Research (MICHR), University of Michigan, Ann Arbor, MI, USA

**Keywords:** Recruitment, social media, recruitment platform, registry, Facebook

## Abstract

Successful social media recruitment requires specific expertise and constant upkeep, placing an inordinate burden on study teams. Over half of the study teams at the University of Michigan (U-M) surveyed about recruitment assistance needs indicated that they wanted to use social media as a recruitment strategy, but lacked the expertise to do so. We thus built a service to centralize social media recruitment across the university. This involved assembling the right expertise, creating a centralized social media profile, creating linkages to other digital recruitment platforms, building the financial structure, and operationalizing the service. So far, we have helped 94 study teams launch social media campaigns on Facebook and Instagram. These campaigns resulted in 1,653,675 users being reached, of which 20,546 users actively showed interest in participating in the corresponding studies. We followed 18 studies further, who reported a total of 345 social media participants as being enrolled, resulting in an average cost-per-contact (CPC) of $8.72 and an average cost-per-enrollee (CPE) of $55.21. The combination of communication expertise, streamlined administrative processes, and linkages to a centralized research participation registry has allowed us to help a large number of study teams seamlessly engage broad and diverse populations.

## Introduction

The social media revolution has altered the communication landscape and has significantly impacted marketing communication. The growing importance of applications like Facebook, YouTube, Instagram, and others in consumers’ lives has an increasing influence on their communication habits. By some accounts, consumers spend an average of 2.5 h/day in the social media realm, and in turn, an increasing share of communication occurs within these social network environments [1]. As a result, organizations have had to evolve and use (a selection of) these new mediums of communication to reach their intended audience [2,3].

The trend to utilize social media as a means to engage potential consumers has spilled over into the clinical and health research recruitment world. A mandate from NIH to expand diversity in clinical trial representation has pushed study teams to recruit and enroll individuals from traditionally underserved and underrepresented populations in research, but what if the patient population that your institution serves doesn’t necessarily lend itself to diversity? That is where using social media recruitment in conjunction with traditional strategies has shown to help recruit diverse populations to clinical studies [4–7].

Successful social media recruitment requires specific expertise and a constant upkeep, placing a large and sometimes inordinate burden on study teams [8]. The ability to create an environment of trust and transparency in computer-mediated communication is imperative. Rules and restrictions enforced by social media platforms, as well as informal emergent norms in online communities, add to the challenge. Study teams at the University of Michigan (U-M) were faced with this dilemma – the potential benefits of social media recruitment versus the large amount of investments and expertise they require. In response to a recruitment needs survey, study teams indicated that over half of them wanted to use social media as a recruitment strategy but lacked the expertise to do so, while almost 70% of these teams indicated that they would utilize social media as a recruitment strategy if someone else created the campaign and monitored the analytics for them. Study teams reported that the large amount of effort and expertise required to set up, promote, and maintain individual social media profiles was burdensome and did not yield the results they hoped.

On the other hand, qualitative interviews with interested participants revealed that the need to discover and follow multiple social media pages to get access to the various participation opportunities available at U-M was burdensome and unrealistic. This decentralized approach to U-M’s social media presence led the institutional leadership to worry that a single negative experience a participant might have with one research lab could color their view of the entire research enterprise.

As the full picture of social media at U-M came into focus, we began to see that the Michigan Institute for Clinical & Health Research (MICHR), U-M’s Clinical and Translational Science Institute (CTSI), was uniquely positioned to solve this increasing fragmentation. MICHR had the templates, resources, and expertise available to lay the foundation for individual study teams. We decided to build a social media recruitment service to centralize social media recruitment, which would enable research teams to efficiently mobilize social media recruitment strategies without having to repeatedly build the required infrastructure. It would also allow a seamless experience for potential participants who wanted to engage with research at U-M through social media.

## Methods

### Assembling the Expertise

MICHR hired a communications coordinator to lead the creation and monitoring of social media campaigns. This coordinator had a marketing background and was primarily focused on creating diverse, engaging, and lay-friendly advertising for identified studies.

### Choosing Facebook and Instagram as the Social Platforms of Choice

From the growing number of social media platforms available, we decided to spearhead our efforts with Facebook and Instagram. As two of the most active and popular social media channels in the world, these platforms have the largest coverage of the populations our study teams were trying to reach, along with robust tools for businesses and organizations. We also decided to focus on targeted paid advertising as it overcomes some of the disadvantages of organic social media recruitment by reaching people beyond just the followers of a page. Targeted, paid advertising allows for the placement of ads directly onto the *newsfeeds* of individual users who appear to meet the targeting criteria for a given study. This user may also see the advertisement in other places including stories, audience networks, and Facebook marketplace.

### Creating a Centralized Facebook Profile

The MICHR team, with the help of the newly hired communication coordinator, then created a centralized social media profile, a UMHealthResearch Facebook page, to consolidate all research-related messages. This page incorporated the official U-M brand, logo, and color palette, as well as research-focused content. The communication coordinator actively maintained this page, posting relevant research content at least three times per week. All social media advertising for studies served was launched from this page. This page served as a single, unified, and trusted source that people interested in health research could use to access U-M’s health research-related messages on social media.

### Establishing Linkages to a Centralized Study Posting Platform

**Fig. 1.**
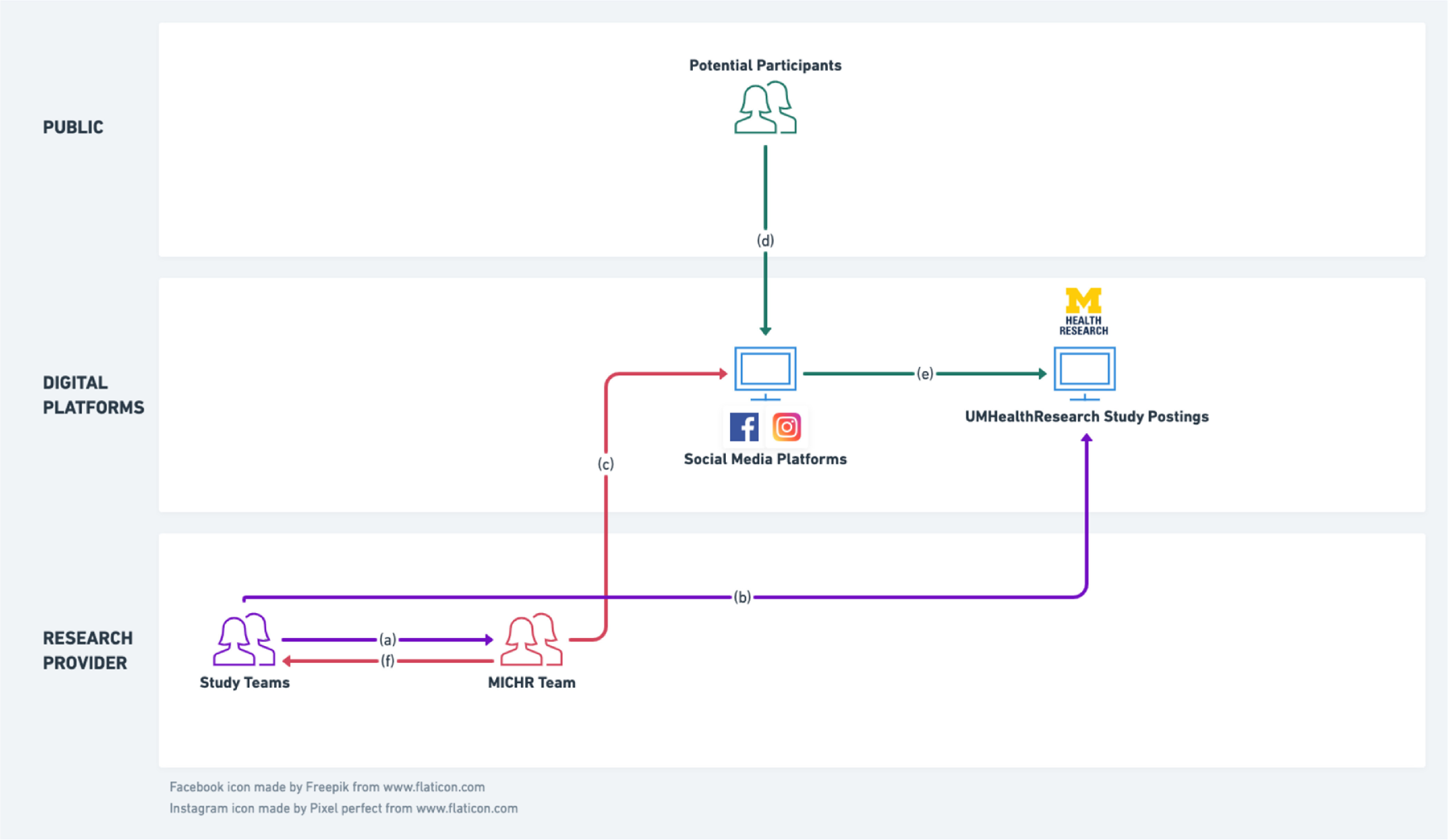
MICHR’s social media recruitment service structure. (a) Study teams approach the MICHR team with social media recruitment requirements. (b) In consultation with the study teams, MICHR designs and posts organic posts on centralized social media profile and uses this centralized profile to launch paid social media campaigns for individual studies. (c) Potential participants engage with these single-source posts and advertisements. (d) Clicking on an advertisement takes a participant to a lay-friendly study posting on our centralized recruitment website, UMHealthResearch.org. (e) MICHR team provides study teams with metrics on effectiveness and conversion.

MICHR maintains a highly successful online recruitment and engagement portal called UMHealthResearch that connects potential volunteers to active research studies [9,10]. This portal offers a lay-friendly description of study offerings, study contact information, and also allows volunteers to be matched to relevant studies. We linked a study’s social media campaign to its individual study description page on UMHealthResearch, providing potential participants a seamless way to hear about a study, learn more about it, and in turn, get into contact with the study team (Fig. 1).

### Building the Financial Structure

This MICHR service is offered to the study teams free of charge, however, study teams are required to pay the social media company (Facebook) for their campaigns. Facebook utilizes a pay-per-click algorithm, which is dependent on the target audience and advertising competitors to reach the audience. In order to avoid excessive costs per click, the service utilizes a lifetime budget, which is set on each campaign. The MICHR team uses Facebook’s recommendation of $50/week dependent on the duration and desires of the study team campaign. Utilizing the lifetime budget ensures teams never spend more than the initial dollar amount designated for their campaign. The UMHealthResearch *business* page is linked to a university credit card that is issued to the communications coordinator. This credit card is charged every month and every time the monthly threshold is met.

### Operationalizing the Service

To facilitate this newly created service, we designed the following process:

1. **Communication and Marketing:** We communicated the launch of this service through various channels including our existing 1:1 recruitment consultations with study teams, presentations at departmental meetings, and email newsletters.
2. **Intake form:** Interested study teams completed a social media request form to provide the necessary information for targeting, budget, and general study information (Appendix A). It should be noted that along with social media, we advised study teams to deploy appropriate complementary recruitment strategies to achieve their goal.
3. **Mock-up ads:** Upon receipt of the intake form, the communications coordinator worked with the study team to create mock-up advertisements (see Appendix B, for example).
4. **IRB approval:** These advertisements were then sent to the IRB for approval.
5. **Campaign Launch:** Upon receiving approval, the social media campaign was launched and monitored by the communications coordinator. Study teams had the ability to turn on, turn off, and make changes to the campaign throughout the duration in real time.
6. **Billing, Reporting, and Analytics:** Weekly analytics were provided by the communications coordinator to the study team for their review. The communications coordinator reconciled the cost-per-click charges with the specific study short code and sent a receipt to the study (see Appendix C, for example).

## Measuring Impact

The metrics we used to measure impact are shown in Table 1.

**Table 1.** Metrics used to measure impact

Metric	Definition	Source
Reach	The number of people who see your ads at least once.	Facebook/Instagram
Link clicks	The number of clicks on links within the ad that led to destinations or experiences, on or off Facebook.	Facebook/Instagram
No. of “I am interested”	The number of participants that showed interest in a research study on UMHealthResearch after engaging with the ads.	UMHealthResearch
No. of contacted	The number of participants originating from social media that study teams had contacted.	Study teams
No. of enrolled	The number of participants originating from social media that study teams had enrolled.	Study teams

### Engagement on Facebook

The communications coordinator provides study teams with weekly analytics stating the reach, results, amount of clicks, click-through rate (CTR), and frequency (see Appendix C, for example). These analytics are provided both at the ad set and ad level. Comments and likes are monitored by the communications coordinator, with the default set to refer any questions to the study team, utilizing contact information found on the UMHealthResearch posting.

### Engagement on UMHealthResearch

In order for the MICHR team to measure the success of a campaign, a pixel (snippet of code) was embedded in UMHealthResarch.org. This pixel allowed us to track the potential participants who have come to UMHealthResearch through Facebook and their activities on the portal. Thus, the MICHR team was able to provide the study teams with a detailed report, including: (a) the views a UMHealthResearch study posting received due to a corresponding social media ad and (b) the number participants that connected to the study team through UMHealthResearch after engaging with the ad.

### Enrolled Participants

As part of our study intake process, study teams were asked to report the number of currently enrolled volunteers (prior to the social media campaign launch). Teams also had to agree to track the number of volunteers who identified the use of social media advertising as a means of connecting to the study. Lastly, study teams had to agree to complete a 6-month post-consultation survey whereby reporting the total number of participants who enrolled in their study due to a social media ad campaign.

## Results

**Fig. 2.**
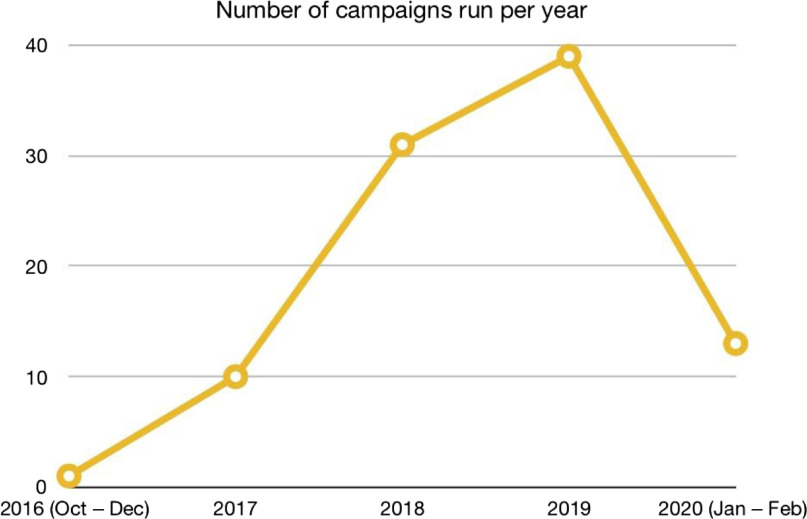
Campaigns run per year.

Since October 2016 through the time of writing (February 2020), we have launched 94 social media campaigns on Facebook and Instagram (Fig. 2). Each campaign was made up of multiple ad sets. In the time period above, we created a total of 500 ad sets. Each ad set had between 1 and 6 ads amounting to a total of 2554 ads.

These campaigns have resulted in 1,653,675 users being reached on these social media platforms, of which 109,633 of these users clicked on an ad we had launched and landed on the corresponding study-specific UMHealthResearch.org posting. Of those, 20,546 users got connected with the study team on UMHealthResearch by clicking the “I’m interested” button for that particular study.

Of the 94 study teams serviced, 18 teams reported back to the MICHR team with (a) the number of participants originating from social media that they had been in contact with and (b) the number of participants originating from social media they had enrolled. Based on these numbers, we calculated the social media cost-per-contact (CPC) and the cost-per-enrollee (CPE) for each study. A breakdown of the high-level study eligibility requirements for these 18 studies, the numbers they reported, and their individual CPC and CPE are shown in Table 2. On the whole, these teams reported that a total of 2637 users originating from social media contacted them for more information about their studies, while 345 participants originating from social media were enrolled. The average CPC across these studies was $8.72 while the average CPE was $55.21.

**Table 2.** List of studies who reported additional recruitment numbers

Campaign	Contacted	Enrolled	CPC	CPE
Study A: target audience of women 25–40 that are wishing to eat healthier.	1044	5	$0.78	$162.98
Study B: target audience of parents of 3–7 years old with stuttering issues.	4	2	$25.00	$50.00
Study C: target audience of parents of 7–12 years old that show symptoms of OCD.	7	0	$14.20	n/a
Study D: target audience of pregnant or new mothers.	491	9	$1.19	$64.72
Study E: target audience of parents and legal guardians of children 6–10 years old.	26	7	$12.38	$45.99
Study F: target audience of adults 18 and older with fibromyalgia.	104	2	$2.69	$139.78
Study G: target audience of parents/legal guardians of 2–5 years old.	10	7	$4.24	$6.06
Study H: target audience of parents/legal guardians of 2–5 years old.	140	113	$5.56	$6.89
Study I: target audience of minorities (identified as Latin, African-American, and Asian) 41 and older iOS users.	87	30	$16.09	$46.67
Study J: target audience of families with children between 8 and 10 years old.	27	21	$7.41	$9.52
Study K: target audience of adults 18 and older with schizophrenia and visual perception issues.	22	4	$9.09	$50.00
Study L: target audience of women 18 and older with physical mobility issues.	16	16	$12.50	$12.50
Study M: target audience of women 18 and older with physical mobility and reproductive issues.	34	30	$11.76	$13.33
Study N: target audience of women 18 and older with physical mobility issues.	15	6	$13.33	$33.33
Study O: target audience of teens and young adults.	70	55	$1.43	$1.82
Study P: targeted audience of males and females within 50 miles of Ann Arbor and 18–40 years old.	89	11	$4.49	$36.36
Study Q: targeted audience of males and females within 50 miles of Ann Arbor and 18–40 years old.	55	14	$7.27	$28.57
Study R: targeted audience of males and females within 50 miles of Ann Arbor and 18–40 years old.	396	13	$7.50	$230.00
Total	2637	345	NA	NA
Average	147	19	$8.72	$55.21

We noticed that studies that had specific inclusion criteria, such as a lab value or diagnosis, generally had a lower amount of enrollees. Additionally, studies that required multiple visits, an invasive procedure, and/or time-consuming study tasks, also had a lower amount of enrollees.

While we advised all study teams to deploy multiple recruitment strategies to achieve their recruitment goal, we did not systematically collect quantitative information on how these approaches compared with social media. We did, however, repeatedly hear that independent of the tangible money spent on recruitment strategies (social media ad spend, printing, etc.), study teams saved a significant amount of time and effort using the centralized social media service. One team shared that “You are definitely our major source of recruitment and I would like to keep this going until we are done with the project;” while another read “I can say that since running the ad we have a lot more traffic on our UMHealthResearch site than ever before.” A multi-site study utilizing social media advertising as one of their strategies offered the following comment: “We are the top recruiting site for this multi-site trial due to our Facebook ads.”

Due to the success of our social media recruitment, we have had multiple institutions and pharmaceutical companies reach out to understand our process in creating these campaigns.

Finally, an unintended consequence of our social media setup has been a strong growth in volunteer profiles being created on UMHealthResearch. In the timeframe in question, 6794 new UMHealthResearch.org profiles have been created by participants who indicated that they learned about the platform from a social media campaign. These volunteers were introduced to the platform because of a particular study being promoted on social media, they then created a profile and signed up to receive future notifications about other studies that they would be eligible for. Although we did not collect demographic participant data for individual studies we serviced, we were able to gain insights into the race and ethnicity make up for the new UMHealthResearch.org profiles created (Table 3). The racial and ethnic makeup of the profiles mirrored that of the state of Michigan’s population.

**Table 3.** Racial and ethnic distribution of UMHealthResearch profiles who indicated that they learned about the platform from a social media campaign

Race	
American Indian or Alaska Native	0.60%
Asian	2%
Black or African-American	10%
Native Hawaiian or other Pacific Islanders	0.10%
White or Caucasian	78%
Two or more races	9%
**Ethnicity**	
Hispanic or Latino	5%
Not Hispanic or Latino	95%

## Conclusion

Social media has the potential to be an impactful tool in the recruitment portfolio of a study team. However, effective social media recruitment needs specific expertise and infrastructure. Through this work, we hope to have surfaced our efforts to create a centralized social media engagement framework that can help study teams enjoy the benefits of efficient and effective social media recruitment without some of the accompanying burdens. Our results indicate that the combination of communication expertise and streamlined administrative processes in tandem with the linkages between social media channels and a centralized research participation registry has allowed us to help a large number of study teams seamlessly engage broad and diverse populations. This broad framework is likely generalizable and warrants investment from research administrators who want to help their teams take efficient advantage of the benefits of social media recruitment.

It must be acknowledged that not all studies are fit for social media recruitment. We have found social media to be most useful for studies that need healthy participants or where there are loose inclusion/exclusion criteria. Feedback from study teams also suggests that social media does not work well for studies that have very narrow inclusion criteria. These studies may be a better fit for in-person recruitment at the point of care, as inclusion criteria involving specific lab values, diagnoses, and results from specific procedures may be difficult to promote to a broad audience. This is not to discourage the strategy for rare diseases or specific condition studies. We have found that this strategy produces positive results where there is a strong social media community around a particular medical topic, such as certain cancers and rare diseases that have a strong presence on social media platforms.

As social media usage evolves, we hope to bring other platforms, such as Snapchat, YouTube, and TikTok into our portfolio, continually evaluating for effectiveness and fit. We want to continually experiment with social media strategies to engage deeper with special populations. Additionally, we hope to create an increasing spectrum of synergies between social media and other recruitment strategies with the goal of providing a seamless experience for research participants while being a centralized recruitment innovation resource for the research enterprise.

## Supplementary material

For supplementary material accompanying this paper visit https://doi.org/10.1017/cts.2020.540. click here to view supplementary material
