# Supplementary material for: Creating a centralized social media recruitment service for research teams at the University of Michigan
Source: J Clin Transl Sci. 2020 Sep 11;5(1):e47. doi: 10.1017/cts.2020.540 (PMC8057388; doi:10.1017/cts.2020.540)
Supplement: Supplementary file 1 [file S2059866120005403sup.zip › S2059866120005403sup002.docx]

Appendix A: Social Media Request Form

1. Do you have IRB approval to use social media as a recruitment tool?
   - Yes
   - No
2. Have you verified your social media request with your IRB staff owner?

(Although we use recycled content from your study materials, some ads may require further review by your IRB staff owner)

- - Yes
  - No

1. Have you had a social media consultation (or discussion) with participation recruitment?
   - Yes
   - No
2. Do you plan to post your study on UMHealthResearch.org?
   - Yes
   - No
3. Please complete the following regarding target information:

| Location |  |
| --- | --- |
| Age |  |
| Gender |  |
| Detailed Targeting | (i.e. support group pages) |
| Short code and person responsible for short code |  |
| Budget | (recommended $50/wk) |
| Duration of Campaign | (recommended 1 month) |
| Start Date of Campaign |  |
| Amount of Images Desired (max. of 6) |  |
| Study HUM# |  |
