# Supplementary figures and images for: Creating a centralized social media recruitment service for research teams at the University of Michigan
Source: J Clin Transl Sci. 2020 Sep 11;5(1):e47. doi: 10.1017/cts.2020.540 (PMC8057388; doi:10.1017/cts.2020.540)

B. Example ads


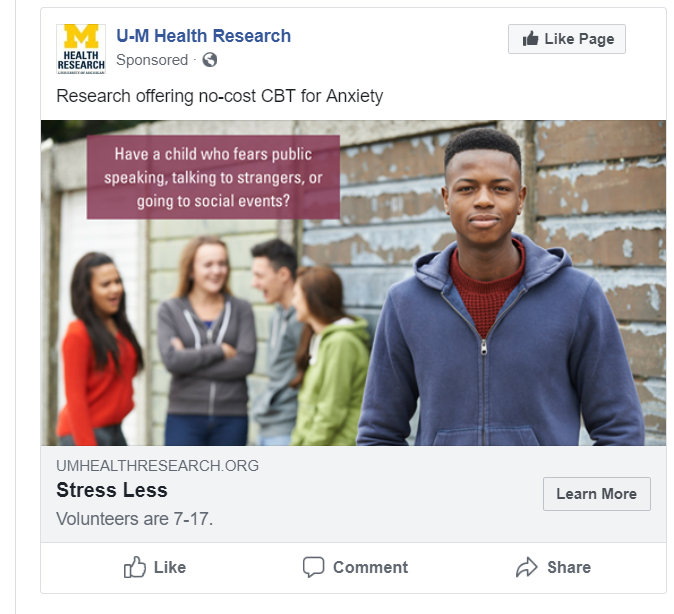

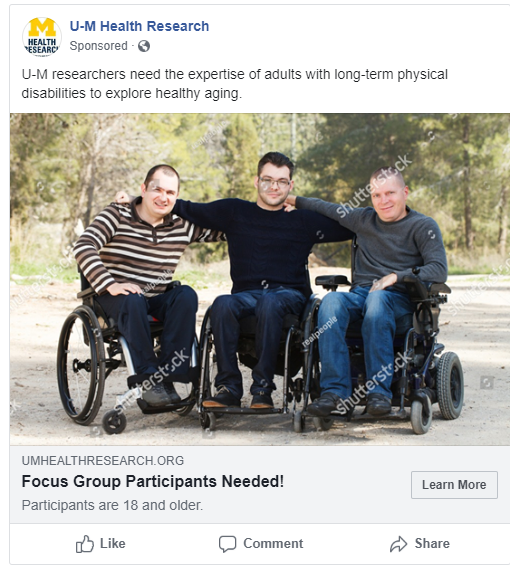

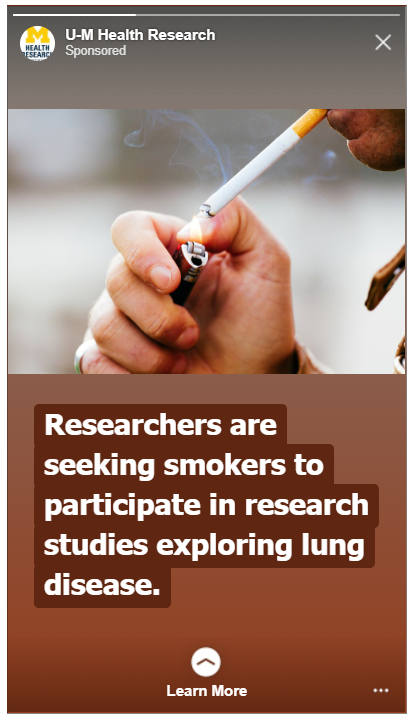

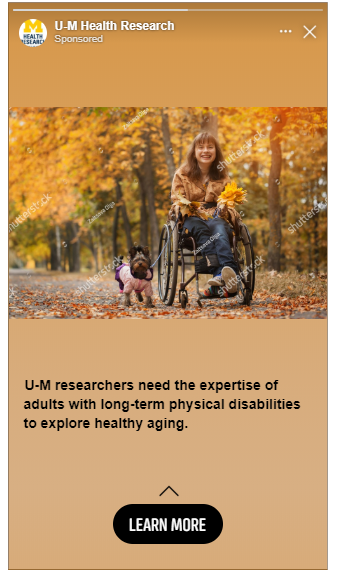

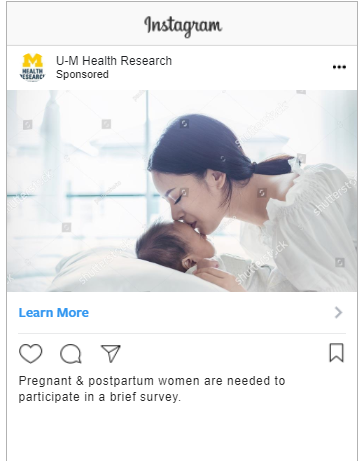

Supplement: Supplementary file 1 [file S2059866120005403sup.zip › S2059866120005403sup001.docx]
